# Supplementary material for: Arbovirus vectors in municipalities with a high risk of dengue in Cauca, Southwestern Colombia
Source: GigaByte. 2022 May 16;2022:gigabyte53. doi: 10.46471/gigabyte.53 (PMC9930565; doi:10.46471/gigabyte.53)
Supplement: Translation

# Translation: Vectores de arbovirus en municipios con alto riesgo por dengue en el departamento del Cauca, Colombia

**Authors:** Catalina Marceló-Díaz, Carlos Andres Morales, Maria Camila Lesmes, Patricia Fuya, Sergio Andres Mendez, Horacio Cadena, Alvaro Ávila-Díaz, Erika Santamaria

**Affiliations:** ^1^ Grupo de Entomología, Instituto Nacional de Salud, Avenida Calle 26 51-20, Bogotá, Colombia; ^2^ Secretaría de Salud Departamental del Cauca, Calle 5 15-57 Barrio Valencia, Popayán, Colombia; ^3^ Universidad de Ciencias Aplicadas y Ambientales, Calle 222 55-37, Bogotá, Colombia; ^4^ Programa de Estudio y Control de Enfermedades Tropicales PECET, Calle 62 No. 52-59, Medellín, Colombia

## Abstract

Aquí proporcionamos nuevos datos inéditos sobre la distribución de 2.383 ejemplares pertenecientes a los géneros *Aedes* spp. y *Culex* sp. (Diptera: Nematocera). Además, se reporta el porcentaje de infestación domiciliaria en el área urbana de tres municipios del departamento del Cauca (Colombia). Este conjunto de datos es de relevancia en salud pública teniendo en cuenta que *Aedes* spp. es capaz de transmitir arbovirus como el dengue, el Zika y el chikungunya, y *Culex* sp. es un conocido vector del virus del Nilo Occidental y de la encefalitis equina venezolana.

## Descripción de los datos

### Introducción

Las arbovirosis como el dengue, el chikungunya, la fiebre amarilla y más recientemente el Zika, se están expandiendo en el mundo, situación que se convierte en una preocupación para la salud pública a nivel internacional [1, 2]. En las Américas, el 2019 fue el año con el mayor número de casos de dengue de la historia con más de 2,7 millones, incluyendo 22.127 casos graves y 1.206 muertes reportadas [3].

Las principales especies de mosquitos implicadas en la transmisión de arbovirosis son *Aedes aegypti* (Linnaeus), *Aedes albopictus* (Skuse) y *Culex quinquefasciatus* (Say), las cuales hacen parte de la familia Culicidae (Diptera: Nematocera). Esta familia cuenta con 3.200 especies reconocidas, y es probable que exista un buen número de especies por descubrir, principalmente en las selvas tropicales, donde se concentra una mayor biodiversidad [4].

El mosquito *Ae. aegypti*, es una especie predominantemente doméstica, que de manera general infesta criaderos naturales o artificiales en las viviendas o sus alrededores. La hembra se alimenta de sangre humana o de animales domésticos [5]. Debido a su estrecha relación con el hombre, es esencialmente un mosquito urbano. En Colombia, se ha registrado en un rango altitudinal va de los 0 a los 2.302 m s.n.m. [6] y se ha reportado también en áreas rurales [7].

Las hembras de *Ae. aegypti* restringen sus hábitos hematófagos a horas diurnas. Sus picos de mayor actividad se encuentran en el amanecer y un poco antes del crepúsculo vespertino, aunque puede picar a cualquier hora del día. El hábito diurno también ha sido registrado en el macho, tiempo en el que copulan y se alimentan de sustancias azucaradas [8, 9]. En condiciones óptimas de disponibilidad de alimento y zonas de oviposición adecuadas, la dispersión media de una hembra de *Aedes* spp. se estima entre 50 y 100 m, lo que limita sus visitas a 2 o 3 viviendas durante su vida adulta [7, 10], aunque se han registrado hembras alimentadas con capacidad para dispersarse hasta 800 m en 6 días [11].

A diferencia de *Ae. aegypti*, el mosquito *Ae. albopictus* (Skuse) se encuentra con mayor frecuencia en regiones forestales y en espacios abiertos con abundante vegetación, propios de zonas suburbanas o rurales. Pueden encontrarse en ambientes con extensa vegetación, aprovechando como criaderos naturales los tocones de bambú, cavidades de árboles, axilas de plantas (bromelias) y depósitos de agua en grietas de las rocas [12]. Cuando cohabita con el *Ae. aegypti* en la zona urbana, puede desarrollarse muy bien en depósitos artificiales como floreros, llantas y latas [13]. Aunque *Ae. albopictus* es antropofílico, el comportamiento hematófago de las poblaciones de esta especie de mosquito puede depender de la disponibilidad de las fuentes alternativas de alimentación (animales domésticos y silvestres) [14].

Finalmente, *Culex quinquefasciatus* (Say), es considerada una especie acentuadamente antropofílica y que se encuentra asociada con mayor frecuencia al hábitat humano tanto urbano como rural en Colombia [15]. Su actividad de picadura se asocia mayormente a hábitos nocturnos, sin embargo, permanece durante el día en el interior de la vivienda [8, 16]. Se distribuye por todo el territorio nacional en alturas que van desde los 0 hasta los 3.000 m s.n.m [15]. Junto con *Ae. aegypti*, los estadios inmaduros de esta especie también se desarrollan en estructuras que sirven de desagües y sumideros de aguas lluvias ubicados en calles y avenidas [17].

El siguiente artículo de datos contiene la descripción y cuantificación de los mosquitos adultos del género Culicidae (Diptera: Nematocera) recolectados con aspiradores Prokopack en viviendas del área urbana de tres municipios con alta incidencia de dengue en el departamento del Cauca (Suroeste Colombia).

### Contexto

Los 2.383 especímenes (529 ocurrencias) de la familia Culicidae en este conjunto de datos son registros inéditos para el departamento del Cauca, Colombia. Los datos fueron obtenidos en el año 2021 por un equipo multidisciplinario conformado por ingenieros geógrafos y ambientales, así como profesionales y técnicos con amplia experiencia en entomología médica.

Los mosquitos fueron recolectados en tres municipios del Cauca, departamento seleccionado por presentar un comportamiento endemo-epidémico para el dengue, es decir, presentación de endemias focalizadas, escenarios variables de transmisión y patrones temporales, estacionales y cíclicos en poblaciones de riesgo.

Los municipios integran el área de estudio de un proyecto de investigación que pretende establecer la relación entre los factores ambientales, biológicos y sociodemográficos que influyen en el aumento de la carga de dengue y su variación espacial; por medio del uso de los sistemas de información geográfica en la construcción de mapas de riesgo.

La información presentada es de utilidad para las autoridades sanitarias, ya que en cada municipio se muestran los barrios donde se concentró una mayor densidad de mosquitos. Por otra parte, la georeferenciación de las capturas permitirá realizar nuevos análisis asociando otras variables entomológicas y ambientales.

## Metodología

### Muestreo

La recolección de mosquitos adultos de la familia Culicidae, se llevó a cabo en los municipios de Piamonte, Patía y Miranda, seleccionados por presentar un alto riesgo de dengue, de acuerdo con el análisis global de clúster de la variable tasa de incidencia por cada 10.000 habitantes en el departamento del Cauca, análisis para el cual se utilizó la técnica High-Low Clustering (Getis-Ord General) en el software ArcGIS^®^ 10.8 (SCR_011081). Posteriormente, se delimitó el muestreo a nivel de barrio con el análisis de densidad de Kernel (Hot spot analysis) con los casos de dengue registrados entre el 2015 y el 2019 en el área urbana de cada municipio.

En total, se muestrearon 935 viviendas en 17 barrios de los tres municipios durante el año 2021. En el municipio de Piamonte y Patía se identificaron tres conglomerados para la transmisión del dengue, por lo cual se muestrearon tres barrios en el primer municipio (*n* = 180 viviendas) y siete barrios en el segundo (*n* = 335 viviendas). Finalmente, en el municipio de Miranda se muestrearon seis barrios de cuatro conglomerados (*n* = 420 viviendas) con incidencia de la enfermedad más alta que la esperada.

### Recolección de mosquitos

Se realizó la inspección entomológica entre las 8:00 y las 17:00 horas, en un tiempo promedio de 10 minutos por vivienda. En cada vivienda se utilizó un aspirador Prokopack ((Figura 1) realizando un barrido en las paredes de las habitaciones (incluyendo muebles y ropa colgada), la sala de estar, el comedor, los baños, la cocina y el patio. Posteriormente, los mosquitos recolectados fueron anestesiados para facilitar su identificación.

**Figura 1.** (A)  Recolección de mosquitos en el intradomicilio con aspirador Prokopack, (B) mosquitos recolectados en una de las viviendas.

### Clasificación de especies y caracterización espacial

La identificación taxonómica y clasificación de las especies *Aedes aegypti*, *Aedes albopictus* y *Culex quinquefasciatus* fue realizada por entomólogos expertos, mediante el uso de claves taxonómicas especializadas como las de Forattini [8] y Rueda [18] (Tabla 1); diferenciando además hembras de machos.

**Tabla 1:** Caracteres entomológicos descriptivos de las tres especies de mosquitos registradas.

*Especie*	*Claves taxonómicas*
*Aedes aegypti*	Diseño doble de liras submediales longitudinales blancas en el *scutum*, el clípeo con escamas blancas (hembras), las escamas separadas en el mesanepimeron y los tarsómeros de la tercera pata o pata posterior con escamas basales blancas [7, 8, 18].
*Aedes albopictus*	Presencia de una sola lira medial longitudinal blanca en el *scutum,* clípeo sin escamas (hembras), las escamas del mesanepimeron en forma de ”v”. Los esternitos abdominales III-V están cubiertos por escamas oscuras [8].
*Culex quinquefasciatus*	Se diferencia de *Aedes* spp. ya que el *scutum* tiene un tegumento marrón (claro u oscuro), densamente cubierto de escamas amarillas [8].

Siguiendo el protocolo para el desarrollo del trabajo de campo [19], las hembras de *Ae. aegypti* una vez identificadas, fueron almacenadas en viales de 0,2 ml para su procesamiento por técnicas de biología molecular (Figura 2). Las demás especies se almacenaron en seco para su posterior ingreso a la colección del Grupo de Entomología, en el Instituto Nacional de Salud (Bogotá, Colombia).

**Figura 2.** Protocols.io protocolo para el desarrollo del trabajo de campo [19]. https://www.protocols.io/widgets/doi?uri=dx.doi.org/10.17504/protocols.io.b5m7q49n

**Tabla 2:** Indicadores entomológicos por municipio.

	Municipio			
Indicador entomológico	Patia	Miranda	Piamonte	Total
Número de viviendas muestreadas	335	420	180	935
N. total de Culicidae	1156	301	926	2383
N. total de mosquitos de *Ae. aegypti* (%)	305 (26.4%)	223 (74.1%)	44 (4.7%)	572 (24.0%)
N. de viviendas positivas para *Ae. aegypti* (% de infestación)	102 (30.4%)	102 (24.3%)	28 (15.5%)	232 (24.8%)
N. de hembras de *Ae. aegypti* (%)	161 (52.8%)	118 (52.9%)	20 (45.4%)	299 (52.3%)
N. de viviendas positivas para hembras de *Ae. aegypti* (%)	73 (71.6%)	75 (73.5%)	15 (53.6%)	163 (70.2%)
N. de machos de *Ae. egypti* (%)	144 (47.2%)	105 (47.1%)	24 (54.5%)	273 (47.7%)
N. de viviendas positivas para machos de *Ae. aegypti* (%)	63 (61.8%)	56 (54.9%)	17 (60.7%)	136 (58.6%)
Relación hembra: macho de *Ae. aegypti*	1.1:1	1.1:1	0.8:1	1.1:1
N. total de mosquitos de *Cx. quinquefasciatus* (%)	831 (71.9%)	76 (25.2%)	868 (93.7%)	1775(74.5%)
N. de viviendas positivas para *Cx. quinquefasciatus* (% de infestación)	121 (36.1%)	49 (11.7%)	108 (60.0%)	278 (29.7%)
N. de hembras de *Cx. quinquefasciatus* (%)	190 (22.9%)	53 (69.7%)	374 (43.1%)	617 (34.8%)
N. de viviendas positivas para hembras de *Cx. quinquefasciatus* (%)	66 (54.5%)	39 (79.6%)	85 (78.7%)	190 (68.3%)
N. de machos de *Cx. quinquefasciatus* (%)	641 (77.1%)	23 (30.3%)	494 (56.9%)	1158(65.2%)
N. de viviendas positivas para machos de *Cx. quinquefasciatus* (%)	105 (86.8%)	13 (26.5%)	91 (84.2%)	209 (75.2%)
Relación hembra: macho de *Cx. quinquefasciatus*	0.3:1	2.3:1	0.7:1	0.5:1
N. total de mosquitos de *Ae. albopictus* (%)	20 (1.7%)	2 (0.7%)	14 (1.5%)	36 (1.5%)
N. de viviendas positivas para *Ae. albopictus* (% de infestación)	20 (6.0%)	2 (0.5%)	5 (2.8%)	27 (2.9%)
N. de hembras de *Ae. albopictus* (%)	9 (45.0%)	2 (100.0%)	8 (57.1%)	19 (52.8%)
N. de viviendas positivas para hembras de *Ae. albopictus* (%)	8 (40.0%)	2 (100.0%)	5 (100.0%)	15 (55.6%)
N. de machos de *Ae. albopictus* (%)	11 (55.0%)	0 (0.0%)	6 (42.9%)	17 (47.2%)
N. de viviendas positivas para machos de *Ae. albopictus* (%)	5 (25.0%)	0 (0.0%)	2 (40.0%)	7 (25.9%)
Relación hembra: macho de *Ae. albopictus*	0.8:1	2.0:0	1.3:1	1.1:1

 A los mosquitos de cada vivienda se les asignó un código el cuál fue asociado con la encuesta sociodemográfica realizada mediante la aplicación ArcGIS^®^ Survey 123. Lo anterior, permitió establecer la posición geográfica del sitio de recolección de los especímenes. Además de los metadatos relacionados con la localización, se obtuvo la altitud a partir de la plataforma de la NASA [20], la cual alberga el Modelo de Elevación Digital (MDE) Global a una resolución de 12,5 metros.

## Validación de datos

Un total de 2.383 mosquitos de la familia Culicidae fueron recolectados en las viviendas inlcuidas en el muestreo: *Aedes aegypti* (*n* = 572), *Aedes albopictus* (*n* = 36) y *Culex quinquefasciatus* (*n* = 1.775). La Tabla 2 presenta el porcentaje de infestación en las viviendas de cada especie por municipio, así como otros indicadores de importancia en la vigilancia entomológica.

Centrándonos en el vector *Ae. aegypti*, la Figura 3 presenta, para cada municipio, el términos de porcentajes, las viviendas negativas para este vector, las viviendas con entre 1 y 5 mosquitos de esta especie y las viviendas con 6 o más *Ae. aegypti*. Cabe destacar que en tres casas de Patía, más de 15 *Ae. aegypti* y el mayor número de adultos colectados en una sola casa fue de 60. En Miranda se encontraron más de 10 individuos en cuatro casas y el mayor número de adultos en una sola casa fue de 20. Por último, Piamonte presentó el número más bajo de adultos por casa, y el número más alto en una sola casa fue siete.

**Figura 3.** Porcentaje de viviendas en las cuales el correspondiente número de adultos de *Ae. aegypti* fue recolectado.

**Tabla 3:** Índices de riqueza y diversidad de especies de mosquitos encontrados en el departamento del Cauca, Colombia.

									Shannon Weiner Index	Simpson’s Dominance
	*fi*	*fi* log *fi*	*fi* log ^2^ *fi*	*Pi*	*Ni* (*ni* −1)∕*n* (*N* −1)	*Pi* log *Pi*	*Pi* ln *Pi*	*Pi* (ln *Pi*)2	*H* = (*N* log *N* − 𝛴 *fi* log *fi*∕*N*) (or) − (*Pi* log *Pi*)	Index *C* = 𝛴
*Ae. aegypti*	572	1577.2305	4349.0492	0.2400	0.0576	−0.1488	−0.3425	0.4888	0.1488	0.0576
*Ae. albopictus*	36	56.0269	87.1948	0.0151	0.0002	−0.0275	−0.0633	0.2655	0.0275	0.0002
*Cx. quinquefasciatus*	1775	5767.3271	18739.1897	0.7449	0.5548	−0.0953	−0.2194	0.0646	0.0953	0.5548
Σ	2383	7400.5845	23175.4337	1	0.6127	−0.2716	−0.6253	0.81895	0.2716	0.6127

Como medida de la heterogeneidad de la comunidad, se utilizaron los índices de diversidad de Shannon y Simpson [21]. El índice de diversidad promedio para los tres municipios osciló entre 0,27 y 0,66 para el índice de Shannon y entre 0,12 y 0,41 para el índice de Simpson. Ambos índices fueron más altos en Patía y más bajos en Piamonte. Gráficamente, la diversidad de ubicaciones se puede visualizar mejor utilizando el índice de Margalef. Para Miranda la muestra es muy diversa, mientras que para los demás grupos se observa una muestra de características diversas. La Figura 4 muestra la distribución y densidad de los mosquitos recolectados en el intradomicilio en el área urbana de los tres municipios.

**Figura 4.** Medidas de diversidad de especies para cada municipio. El índice de Shannon está representado por barras azules, el índice de Simpson está representado por barras verdes y el índice de Margalef está representado por barras rojas.

 En todas las capturas de mosquitos se utilizaron formatos electrónicos previamente diseñados en Survey123 (ESRI) para registrar la información de la recolección en el campo y las coordenadas geográficas de las viviendas. Expertos entomólogos realizaron la identificación taxonómica. Los registros del conjunto de datos fueron verificados y confirmados uno por uno.

El mayor índice de diversidad se registró para *Ae. aegypti* (0,1488), seguido de *Cx. quinquefasciatus* (0.0953), y los valores más bajos se registraron en *Ae. albopictus* (0.0275). Se estimó el índice de dominancia de todos los mosquitos muestreados en tres municipios diferentes (Cuadro 3); los valores más altos se registraron en *Cx. quinquefasciatus* (0,5548) seguido de *Ae. aegypti* (0.0576) y menos en *Ae. albopictus* (0,0002). Estos resultados son similares a los de otros estudios previos [8].

La Figura 5 muestra el mapa de ubicación y distribución de los especímenes de Culicidae colectados en el área urbana de los municipios de Patía (Figura 5A), Miranda (Figura 5B) y Piamonte (Figura 5C), diferenciados por género y número de especímenes por vivienda. La categoría taxonómica Aedes incluye la especie transmisora del dengue *Ae. aegypti* y *Ae. albopictus*, mientras que la categoría Culex incluye la especie *Cx. quinquefasciatus*, que también está implicado en la transmisión de arbovirosis.

**Figura 5.** Distribución y densidad de culícidos recolectados en el área urbana de los tres municipios del departamento del Cauca (Colombia) incluidos en el estudio.

 Sólo Aedes spp. y *Cx. quinquedasciatus* fueron recolectados con el método de muestreo utilizado (aspiradores Prokopack). Para estudios futuros, se deben utilizar métodos de muestreo complementarios o técnicas de monitoreo para mosquitos adultos (por ejemplo, ovitrampas, trampas para mosquitos BG-SENTINEL). Antes de depositar un espécimen en la colección, los curadores de la colección del Grupo de Entomología revisan la información asociada con el espécimen; es decir, la localidad, coordenadas geográficas, sexo, etapa de desarrollo y taxonomía. La información mínima requerida para incluir un espécimen en la colección está relacionada con el Darwin Core estándar y es la misma que la información mínima requerida para la publicación en el Global Biodiversity Information Facility (GBIF).

## Potencial de reutilización

El conjunto de datos de mosquitos aquí presentado, así como la información resumida en la tabla y las figuras constituyen un recurso valioso para las autoridades de salud encargadas de la vigilancia entomológica y control de arbovirosis en la Región y específicamente en el departamento del Cauca.

La base de datos puede ser usada por la comunidad científica interesada en el estudio de las enfermedades transmitidas por vectores en la construcción de modelos de predicción de la distribución de las especies transmisoras de arbovirosis. Para mejorar la accesibilidad y la posibilidad de re-uso de la información, los datos fueron incluidos en la plataforma del Global Biodiversity Information Facility. El *Ae. aegypti* y *Ae. albopictus* base de datos albopictus también puede ser útil para proyectos similares en otros lugares. Sugerimos a otros que hagan que sus datos estén disponibles de manera similar.

## Data Availability

El conjunto de datos presentado en este artículo se encuentra disponible en el repositorio del Global Biodiversity Information Facility [22].

## Nota del editor

Este documento es parte de una serie de artículos de publicación de datos que trabajan con GBIF y cuentan con el apoyo de TDR, el Programa Especial para la Investigación y Capacitación en Enfermedades Tropicales, alojado en la Organización Mundial de la Salud [23].

## Lista de abreviaturas

*NASA*: National Aeronautics and Space Administration; MDE: modelo digital de elevación; ESRI: environmental systems research institute.

## Aprobación ética y consentimiento de participación

Este trabajo fue aprobado por el Comité de Ética y Metodología de la Investigación del Instituto Nacional de Salud, Bogotá, Colombia (código proyecto: CEMIN 13-2019).

## Consentimiento para la publicación

No aplica.

## Financiación

Este trabajo fue financiado por el Instituto Nacional de Salud, Bogotá, Colombia (CEMIN 13-2019), la Secretaría de Salud Departamental del Cauca, la Universidad de Ciencias Aplicadas y Ambientales U.D.C.A. y el Ministerio de Ciencia, Tecnología e Innovación de Colombia (código proyecto: 210484467217).

## Contribuciones de los autores

CM y ES conceptualizaron el estudio. CAM, PF, ES y MCL generaron los datos de campo. CM, ES, MCL y SM redactaron el manuscrito. PF, HC, CAM y AAD revisaron, corrigieron y abrobaron la versión final del manuscrito.

## References

[1] BhattS, GethingPW, BradyOJ The global distribution and burden of dengue. Nature, 2013; 496: 504–507. doi:10.1038/nature12060.23563266PMC3651993

[2] Organización Panamericana de la Salud (OPS). Directrices para el diagnóstico clínico y el tratamiento del dengue, el chikunguña y el zika. Washington DC: OPS, 2022; ISBN: 978-92-75-32487-5.10.26633/RPSP.2022.82PMC929938835875317

[3] Organización Panamericana de la Salud (OPS), Organización Mundial de la Salud (OMS). Actualización Epidemiológica. Dengue. 11 de Noviembre de 2019. Washington DC: OPS/OMS, 2019; https://www3.paho.org/hq/index.php?option=com_docman&view=download&category_slug=dengue-2158&alias=50965-11-de-noviembre-de-2019-dengue-actualizacion-epidemiologica-1&Itemid=270&lang=es. Accessed 18 Februay 2022.

[4] FosterWA, WalkerED. 12 - Mosquitoes (Culicidae). In: MullenG, DurdenL (eds), Medical and Veterinary Entomology. Elsevier, 2002; doi:10.1016/B978-012510451-7/50014-1.

[5] SuazaJD, BarajasJ, GaleanoE Criaderos naturales para *Aedes aegypti* (Linnaeus, 1762) y *Aedes albopictus* (Sus, 1895) vectores de arbovirus en la ciudad de Medellín (Antioquia, Colombia). Boletín del Museo Entomológico Francisco Luis Gallego, 2013; 5: 18–24.

[6] RuizF, GonzálezA, VélezA Presencia de *Aedes (Stegomyia) aegypti* (Linnaeus, 1762) y su infección natural con el virus del dengue en alturas no registradas para Colombia. Biomédica, 2016; doi:10.7705/biomedica.v36i2.3301.

[7] NelsonMJ. *Aedes aegypti*: Biología y ecología. Organización Panamericana de la Salud, 1986; https://iris.paho.org/handle/10665.2/28513. Accessed 18 February 2022.

[8] ForattiniOP. Principais mosquitos de importância sanitária no Brasil. Cadernos de Saúde Pública, 1995; doi:10.1590/S0102-311X1995000100027.

[9] SalvatellaR. *Aedes aegypti*, *Aedes albopictus* (Diptera, Culicidae) y su papel como vectores en las Américas. La situación de Uruguay. Rev. Med. Uruguay, 1996; 12: 28–36.

[10] Ministerio de la Protección Social, Instituto Nacional de Salud, Organización Panamericana de la Salud. Gestión para la Vigilancia Entomológica y Control de la Transmisión del Dengue. 2011; https://www.paho.org/col/dmdocuments/Entomologia_DENGUE.pdf. Accessed 21 February 2022.

[11] HonórioNA, CostaW, LeitePJ Dispersal of Aedes aegypti and Aedes albopictus (Diptera: Culicidae) in an urban endemic dengue area in the state of Rio de Janeiro, Brazil. Mem. Inst. Oswaldo Cruz, 2003; 98: 191–198. doi:10.1590/s0074-02762003000200005.12764433

[12] GomesA, ForattiniOP, KakitaniI Microhabitats de *Aedes albopictus* (Skuse) na região do Vale do Paraíba, Estado de São Paulo, Brasil. Rev. Saúde Pública, 1992; 26: doi:10.1590/S0034-89101992000200007.1307425

[13] NovakR. The asian tiger mosquito, *Aedes albopictus*. In: Center for Vector Biology Rutgers. Wing Beats. 1992; http://vectorbio.rutgers.edu/outreach/species/sp8.htm. Accessed 21 February 2022.

[14] Rúa-UribeGL, Suárez-AcostaC, RojoRA. Implicaciones epidemiológicas de Aedes albopictus (Skuse) en Colombia. Rev. Fac. Nac. Salud Pública, 2012; 30: 328–337.

[15] SalazarMJ, MoncadaLI. Ciclo de vida de *Culex quinquefasciatus* Say, 1826 (Diptera: Culicidae) bajo condiciones no controladas en Bogotá. Biomédica, 2004; 24: 385–392. doi:10.7705/biomedica.v24i4.1288.15678802

[16] Rojas-MogollónMA, Hernández-NeutaGE, Moncada-ÁlvarezLI Actividad de picadura de *Culex quinquefasciatus* (SAY, 1863) en Bogotá, Colombia. Rev. Fac. Med., 2013; 61: 261–266.

[17] Ceron-HernandezVA, PeñaMR, AlexanderN. Randomized trial of a modification of rainwater street catch basins to physically control the *Aedes aegypti* dengue mosquito vector. Ing. Univ., 2020; 24: doi:10.11144/Javeriana.iued24.rtmr.

[18] RuedaLM. Pictorial keys for the identification of mosquitoes (Diptera: Culicidae) associated with dengue virus transmission. Zootaxa, 2004; 589: doi:10.11646/zootaxa.589.1.1.

[19] SantamariaE, Muñozp, MarcelóC. Indoor active search for adult *Aedes* sp. and *Culex* sp. mosquitoes. protocols.io, 2022; doi:10.17504/protocols.io.b5m7q49n.

[20] National Aeronautics and Space Administration (NASA). ASF Data Search Vertex. https://search.asf.alaska.edu/#/. Accessed 15 June 2021.

[21] Senthamarai SelvanP, JebanesanA, ReethaD. Entomofaunal diversity of tree hole mosquitoes in Western and Eastern Ghats hill ranges of Tamilnadu, India. Acta Trop., 2016; 159: 69–82. doi:10.1016/j.actatropica.2016.03.029.27016014

[22] Marceló DíazC, MoralesCA, FuyaOP Registros de dípteros asociados al proyecto: Estratificación espacial del dengue basado en la identificación de factores de riesgo en el departamento del Cauca. v1.3. Instituto Nacional de Salud. Dataset/Occurrence, 2022; doi:10.15472/dxbowv.

[23] Vectors of human disease series. GigaByte. 2022; 10.46471/GIGABYTE_SERIES_0002.
